# Exploring Neurofunctional Phase Transition Patterns in Autism Spectrum Disorder via Thermodynamics Parameters

**DOI:** 10.3390/e28050567

**Published:** 2026-05-19

**Authors:** Dayu Qin, Yuzhe Chen, Ercan E. Kuruoglu

**Affiliations:** Institute of Data and Information, Tsinghua Shenzhen International Graduate School, Tsinghua University, Shenzhen 518055, China; tdy22@mails.tsinghua.edu.cn (D.Q.);

**Keywords:** spectral core entropy, dynamic functional connectivity, thermodynamics framework, brain network, autism

## Abstract

Designing informative descriptors for time-varying complex networks is important for characterizing structural reconfiguration in evolving graph data. This paper introduces a thermodynamics-inspired framework for dynamic graph analysis, centered on Spectral Core Entropy (SCE), node energy, internal energy, and a temperature-like reconfiguration index. These quantities provide a compact representation of how graph organization changes over time. We apply this framework to resting-state fMRI data from autism spectrum disorder (ASD) and control subjects. At the event level, the temperature index shows a statistically significant but modest association with low-SSIM reconfiguration events, indicating that it serves as a weak yet reproducible marker of rapid network change. On controlled synthetic dynamic graphs, the framework exhibits regime-dependent sensitivity: spectral-core change is more informative under rewiring, whereas the temperature index is more informative under gain modulation. At the node level, node energy highlights regional differences between ASD and control groups, providing interpretable neuroscientific context for dynamic brain connectivity. Overall, the proposed framework provides a promising and computationally tractable approach for characterizing reconfiguration patterns in dynamic brain networks and other evolving complex systems.

## 1. Introduction

The activity of the resting state of the human brain cannot be described as random. Instead, there are governing rules of brain activity at different time scales, ranging from a few seconds to even several years (developmental). It has been discovered that functional connectivity in the resting state has several cognitive phenotypes [1] and that spontaneous brain activities are highly organized into hierarchical structures. This brings forward the study of brain states and brain-state transitions. As defined in [2], brain states are recurring brain configurations that emerge from physiological or cognitive processes. It is a building block of neural dynamic models, advancing the understanding of brain activities. The study of brain-state transitions can facilitate sleep stage detection [3], and the differences between brain-state compositions can distinguish task and rest brain activities [1]. The occupancy profile of the brain state can serve as identifiers of individual differences and biomarkers of neurological disorders (ADHD [4], schizophrenia [5], and major depressive disorder [6]).

Despite substantial progress, detecting and quantifying brain-state transitions remains challenging. The concept of “brain state” is operationalized heterogeneously across subfields and timescales, and the observable dynamics depend on modality-specific temporal resolution and the targeted analytic scale [7].

In current studies, there are several ways to categorize brain signals into brain states. Brain signals can be categorized according to biological definition (e.g., brain wave, phase synchronization, potential distribution). Based on different functional networks in the brain, previous work defines brain states to be composed of different sub-states and can be linear combinations of them [3]. However, such definitions are not generalizable, as the biological definition can be flawed, which can limit the emergence of novel findings.

There are also data-driven definitions of brain states. There are studies that perform classification or clustering to identify groups of similar brain configurations. Critical findings have also been made using hidden Markov models (HMMs) [3,8]. However, there are also clear disadvantages of clustering algorithms. For instance, the K-Means algorithm requires the number of clusters to be known or defined beforehand [9]. For algorithms such as DBSCAN, not all data samples (time instants) are clustered [10]. Previous studies have also used matrix distance to quantify the change in brain-connectivity networks [11]. However, such metrics may not explicitly capture graph-structural reconfiguration, which is an important trait of brain activities.

Graphs are powerful tools for dynamic brain-connectivity analysis, since they are suitable for representing co-functioning activities in the brain [12]. Graph signal processing (GSP) further enhances the analysis of network data [13]. GSP methods have been devised to detect robust graph signals against noise [14] and boost computational efficiency [15], adding potential to this field. Previously, GSP has also been used to analyze time-varying financial networks and prediction of financial market crash [16]. GSP has a wide range of applications for brain-network analysis. Huang et al. [17] decompose brain signals according to different levels of smoothness (or rapidness) using graph spectral operations and discovered the correspondence between brain-network activities and graph frequencies. Preti et al. [18] leverage the structure–function coupling implication of graph frequency components, linking brain functional activities with neural architectures through graph metrics at the node and edge level. However, these methods construct graphs based on brain regions while the time information is collapsed in the correlation calculation step, hindering the detailed analysis of brain- state distributions across time. However, there is a lack of an effective summary indicator that helps depict the change in graph structures and transitions in brain states.

Given the research gap mentioned above, a quantitative representation that better captures the change in brain-connectivity structure is needed to identify brain-state transitions. We introduce a thermodynamic framework to measure the structural change in dynamic brain connectivity.

Informed by a recent interdisciplinary study on the combination of physics and neuroscience, an increasing emphasis on the critical role of thermodynamics and information theory in understanding neural dynamics and brain-state transitions [19,20,21,22,23] has emerged. These frameworks suggest that brain functions are constrained by energy efficiency and governed by principles closely aligned with non-equilibrium thermodynamics [22,23]. Concepts like free-energy minimization, which integrates physical and information-theoretic entropies, offer a compelling model for organizing and transitioning between brain states [21]. This perspective posits that the brain’s hierarchical processing architecture operates within thermodynamic principles to minimize metabolic costs while maximizing computational efficacy [19,20]. By applying thermodynamics principles to dynamic connectivity networks, our proposed framework aims to provide a novel method for quantifying brain-state transitions, enhancing our understanding of how neuronal structures support complex cognitive functions across various states.

In this paper, we define a brain state as a category of similar instantaneous connectivity patterns. We also assume that the change in brain connectivity should be continuous within each brain state, but we expect drastic transitions between brain states. Hence, we will aim to detect the transition points, giving us a continuous brain state in the interval between two transition points. To evaluate the effectiveness of this new metric, we apply it to resting-state functional magnetic resonance imaging (fMRI) data to study brain-state transitions for autism versus control subjects.

The concept of entropy proposed within the framework of complex networks is commonly considered a measure of network complexity [24]. Thermodynamics-inspired ideas have already been used in complex-network analysis to define entropy, internal energy, free energy, and related descriptors for time-evolving, biological, and financial networks [25,26,27]. In this work, we adopt a thermodynamics-inspired graph-information framework to quantify reconfiguration in dynamic brain connectivity. Since entropy computation is closely related to the Laplacian spectrum, we further introduce a tractable approximation that preserves the dominant structural information while reducing computational cost.

Throughout this paper, the terms “phase transition” and “temperature” are used in a descriptive sense for dynamic graphs. By “phase transition” we mean brief intervals of rapid, system-wide reconfiguration of brain functional connectivity. The quantity *T* is defined as the finite-difference ratio T:=ΔU/ΔS between successive windows, which indexes the intensity of reconfiguration between successive windows. In this analogy, micro-states correspond to eigenmodes, entropy is computed from their normalized weights, and SCE retains the dominant modes for computational tractability.

### Contributions

- We propose a thermodynamics-inspired representation for time-varying functional graphs using internal energy, Spectral Core Entropy (SCE), node energy, and a discrete temperature index T:=ΔU/ΔS, providing a unified description of graph interaction mass, spectral reconfiguration, and their coupled changes over time.
- We introduce SCE as a computationally efficient spectral descriptor that retains the top-α normalized-Laplacian eigenvalues to track meso-scale structural reconfiguration, and we clarify its relation to full-spectrum von Neumann entropy through alignment and renormalization analyses.
- We design an event-level detection protocol that links thermodynamic reconfiguration markers with low-SSIM structural changes and evaluates them using lag-aware, threshold-free discrimination and permutation-based significance testing.
- On synthetic dynamic graphs and resting-state fMRI data, we show that the proposed quantities capture complementary aspects of network change: spectral-core variation is more sensitive to topology-dominated reconfiguration, the temperature index is more sensitive to strength-dominated reconfiguration, and node energy highlights interpretable brain regional differences between the ASD and control groups.

## 2. Thermodynamics Framework for Graph Data

In this section, we introduce the innovative concepts of Spectral Core Entropy and node energy, which allow us to derive the internal energy and temperature-like reconfiguration index of a graph. This leads to the development of a novel thermodynamics-inspired framework for complex networks.

### 2.1. Preliminary Constructions

For an undirected graph G(V,E) with the node set *V* and edge set *E*, its adjacency matrix *A* can be defined as follows:

(1) $$A_{ij}=\left\{\begin{array}{cc} 1, & (i,j)\in E\\ 0, & \;\mathrm{else},\; \end{array}\right.$$

where *D*, its degree matrix, is a diagonal matrix and can be expressed as

(2) $$D_{ik}=\left\{\begin{array}{cc} \sum_{j}A_{ij}, & i=k\\ 0, & \;\mathrm{else}\;. \end{array}\right.$$

The Laplacian matrix *L* of the graph is the difference between the degree and adjacency matrices, i.e., L=D−A. *L* can be further normalized, leading to the normalized Laplacian matrix $\tilde{L}=D^{-1/2}\left(D-A\right)D^{-1/2}$. Each element in the matrix can be expressed as

(3) $${\tilde{L}}_{ij}=\left\{\begin{array}{cc} 1 & \;\mathrm{if}\;i=j\;\mathrm{and}\;d_{i}\neq 0\\ -\frac{1}{\sqrt{d_{i}d_{j}}} & \;\mathrm{if}\;i\neq j\;\mathrm{and}\;(i,j)\in E\\ 0 & \;\mathrm{otherwise}.\; \end{array}\right.$$

The eigenvalues $\lambda_{i}$ of the normalized Laplacian matrix have the property $\sum_{i=1}^{\left|V\right|}{\tilde{\lambda }}_{i}=\mathrm{Tr}\left[\tilde{L}\right]=|V|,i=1,2,\ldots ,|V|$.

In our work, we use a thermodynamics-inspired representation of the complex network as a graph-level accounting framework. That is to say, we assume the system will occupy *V* micro-states with a certain probability distribution. The probability of the system occupying a micro-state will be $p_{i}={\tilde{\lambda }}_{i}/\sum_{s=1}^{\left|V\right|}{\tilde{\lambda }}_{i}={\tilde{\lambda }}_{i}/\left|V\right|$, where ${\tilde{\lambda }}_{i},i=1,2,\ldots ,\left|V\right|$ represents the eigenvalues of the normalized Laplacian matrix. Then, the Boltzmann entropy of the system can be written as the following:

(4) $$S_{B}=-k_{B}\sum_{i}^{N}p_{i}\ln p_{i}=-k_{B}\sum_{i}^{N}\frac{{\tilde{\lambda }}_{i}}{\left|V\right|}\ln\frac{{\tilde{\lambda }}_{i}}{\left|V\right|}$$

where $k_{B}$ is the Boltzmann constant.

### 2.2. Spectral Core Entropy (SCE)

In previous work [28], the entropy is approximated using Taylor expansion of ln(x) at x=1. The linear approximated form of $-\ln\frac{{\tilde{\lambda }}_{i}}{\left|V\right|}$, which is $\left(1-\frac{{\tilde{\lambda }}_{i}}{\left|V\right|}\right)$, has significant advantages when calculating the upper and lower bounds of entropy. However, from the micro-state probability $p_{i}={\tilde{\lambda }}_{i}/\left|V\right|$, we can see that when |V| is large, the value of $p_{i}$ tends to be closer to 0. This is also observed in practical experiments when there are over hundreds of nodes. Therefore, it is necessary to propose a more accurate approximation method that better reflects reality.

For a complex network that can be represented by a graph, we consider the eigenvalues of its Laplacian matrix to characterize structural properties in the frequency domain. Within this thermodynamics-inspired network representation, these eigenvalues correspond to micro-state-like spectral components that contribute to the system’s structural complexity.

For large-scale networks, calculating all eigenvalues is often impractical or unnecessary. If only the primary states within the thermodynamic system are considered and we ignore the Boltzmann constant, an approximate value of entropy, the Spectral Core Entropy, can be designed as the following:

(5) $$S=-\sum_{i}^{\alpha }p_{i}\ln p_{i}=-\sum_{i}^{\alpha }\frac{{\tilde{\lambda }}_{i}}{\left|V\right|}\ln\frac{{\tilde{\lambda }}_{i}}{\left|V\right|}.$$

where α represents the top-α largest eigenvalues and is an adjustable hyperparameter.

Since the Laplacian matrix is a positive semi-definite symmetric matrix, we can utilize the Lanczos algorithm to solve for the top-α largest eigenvalues. The Lanczos algorithm is a Krylov subspace method used to efficiently approximate the largest eigenvalues of the (normalized) Laplacian, which we employ to compute SCE with reduced cost and the efficiency of the Lanczos algorithm is higher than performing eigenvalue decomposition directly on the entire matrix, especially when dealing with large-scale problems [29].

By focusing on the top-α largest eigenvalues, we retain the dominant spectral components that contribute most strongly to the graph’s structural variation over time, while significantly reducing computational complexity. In this thermodynamics-inspired representation, these leading components summarize the most influential part of the spectral mass and therefore provide a tractable approximation for tracking meso-scale reconfiguration. Accordingly, SCE is intended as a computationally efficient network-information descriptor rather than as a literal thermodynamic entropy of a physical particle system.

### 2.3. State Energy, Internal Energy, and Temperature

#### 2.3.1. Node Energy

We have obtained an approximation of the system’s entropy. To complement this spectral quantity, we next introduce node-level and graph-level energy-like descriptors within the same thermodynamics-inspired representation. Here, node energy is not intended as a physical energy of brain tissue or of a literal particle system. Rather, it is introduced as a locality-based graph quantity to record how the interaction mass is distributed across the nodes. Using this thermodynamics-inspired locality analogy, we define the node energy as follows:

(6) $$U_{i}=\sum_{j}\frac{1}{d_{i}+d_{j}}d_{i}\varepsilon_{ij}$$

where $d_{i}$ is the degree of node *i*, *j* is the set of neighbor nodes of node *i* and $\varepsilon_{ij}$ is the edge between node *i* and *j*. Since we use the adjacency matrix, $\varepsilon_{ij}=1$.

In this formulation, edges carry the graph-level interaction mass that will later be summarized as internal energy, and node energy allocates this mass locally according to the relative connectivity of a node and its first-order neighbors. The degree term is therefore used as a proxy for local participation in graph interactions, rather than as a claim that graph degree is identical to physical energy. According to the principle of locality, node-level energy in our setting depends only on the node and its first-order neighbors (nearest-neighbor interactions) [30]. This locality-based design yields a simple node-level descriptor that emphasizes how strongly a node is engaged in the surrounding connectivity pattern. For this reason, we refer to it as node energy.

#### 2.3.2. Internal Energy

Within this graph-level accounting framework, the internal energy is obtained by summing the node-level interaction allocations across the network. This gives the following expression for graph internal energy:

(7) $$U=\sum_{i}^{N}U_{i}=\sum_{i}^{N}\sum_{j}\frac{1}{d_{i}+d_{j}}d_{i}\varepsilon_{ij}=\left|E\right|.$$

In our framework, the resulting internal energy is given by the total number of edges in the graph. This should be interpreted as a graph-level interaction accounting result: each edge contributes one unit of interaction mass within the present construction, while node energy distributes this mass locally across incident nodes. That is to say, in this thermodynamic-inspired framework we constructed, the total number of nodes does not directly impact the total internal energy of the system. The designed energy focuses more on the connections between nodes and edges. However, the degree information of the nodes is included in the node energy, which can serve as a node characteristic. This can help us analyze specific node behaviors within this overall framework.

*Modeling note.* In this work, “isolated” refers to the *graph-level accounting abstraction* within a short sliding window: the node set (brain regions) is fixed and the edges carry interaction energy inside this set. Thus internal energy is determined solely by within-graph edge changes, without invoking metabolic closure of the biological system. Nodes and edges are interpreted through a particle-like/ interaction-like analogy only for the purpose of coarse-grained graph-level representation.

#### 2.3.3. Temperature

Inspired by the thermodynamic relation between energy and entropy, we introduce a temperature-like index to characterize reconfiguration between adjacent graph windows. In our setting, *U* and *S* are graph-level accounting quantities rather than physical-state variables of an equilibrium thermodynamic system. At each time window, $U_{t}$ and $S_{t}$ characterize the current state of the time-varying graph, while the temporal aspect of the framework is captured through the discrete comparison between adjacent graph states. Therefore, the following relation is used as an operational analogue for dynamic graph reconfiguration rather than as a literal equilibrium law of the biological brain [28,31]:

(8) dU⩽TdS+δw.

Neglecting external work and adopting the corresponding formal inspiration, we write that

(9) dU=TdS.

which motivates the discrete reconfiguration index

(10) $$T=\frac{\Delta U}{\Delta S}.$$

In our framework, for two consecutive time points t=n−1 and t=n, the temperature can be represented in the following discrete form:

(11) $$T_{n}=\left\{\begin{array}{c} 0,n=1\\ \frac{U_{n}-U_{n-1}}{S_{n}-S_{n-1}},n⩾2 \end{array}\right.$$

where $U_{n}$, $U_{n-1}$, $S_{n}$, and $S_{n-1}$ are the values of internal energy and Spectral Core Entropy at t=n and t=n−1, respectively.

For each graph window, we can thus compute the network’s SCE, node energy, internal energy, and temperature-like reconfiguration index. Together, these quantities define the thermodynamics-inspired descriptive framework used in the remainder of the paper.

Although we use temperature terminology here, we use $T_{n}$ only as a dimensionless reconfiguration index between adjacent windows rather than as an equilibrium temperature. No claim of thermodynamic equilibrium, reversibility, or metabolic closure is made for the biological brain. The sign of $T_{n}$ indicates only the relative direction of change between adjacent windows ($T_{n}>0$ when $\Delta U_{n}$ and $\Delta S_{n}$ covary; $T_{n}<0$ when they move in opposite directions), but it is not assigned a direct classical–thermodynamic interpretation in our event-analysis setting. Accordingly, the present framework is intended for network-information analysis of dynamic graph reconfiguration rather than for literal thermodynamic inference about the biological brain.

Next, we will observe the practical utility of our designed thermodynamic framework in our experiments. These definitions provide a thermodynamics-inspired descriptive framework for analyzing dynamic graph reconfiguration and motivate the experimental evaluation in the following sections.

### 2.4. Mapping Between Classical Thermodynamics and Graph-Information Quantities

We summarize the working analogy between classical thermodynamic terms and their graph-information counterparts used throughout this paper in Table 1. It is intended as a descriptive analogy for dynamic graphs, not as a claim of biological equilibrium.

### 2.5. Rationale and Integration of Metrics

We view *U* as the within-graph interaction mass (sum of edge weights) computed on a fixed parcellation and a within-window snapshot—an “isolated” accounting abstraction rather than a claim of metabolic closure. *S* is the von Neumann entropy of the normalized Laplacian, where eigenmodes are micro-states and their normalized eigenvalues $p_{i}$ define the state probabilities. SCE retains only the top-α eigenvalues that concentrate the dominant spectral mass driving integration–segregation, providing a tractable surrogate for *S* that preserves its temporal reconfiguration trends in practice. The “temperature” T:=ΔU/ΔS is used solely as a dimensionless reconfiguration index between consecutive windows—not an equilibrium temperature nor evidence of reversibility—whose magnitude reflects energetic change per unit spectral change. Neuroscientifically, *U* indexes co-fluctuation strength, *S*/SCE summarizes spectral dispersion, node energy highlights hub engagement, and |T| marks abrupt network reconfiguration; SSIM between successive adjacency matrices serves as an orthogonal, non-thermodynamic baseline for event evaluation.

The integration plan is shown as following:

(1) Macro trends: trajectories of U,S,T, across windows;

(2) Event detection: concordance between *T* spikes and SSIM troughs indicates sharp transitions;

(3) Complementarity: we quantify overlap and distinctiveness using (i) lag-aware, threshold-free AUROC for |T| vs. |ΔSCE| on low-SSIM events (Section 5.4) and (ii) dynamic alignment between ΔVNE and ΔSCE (Appendix D).

## 3. Brain-Signal Analysis Procedure

**Figure 1:**
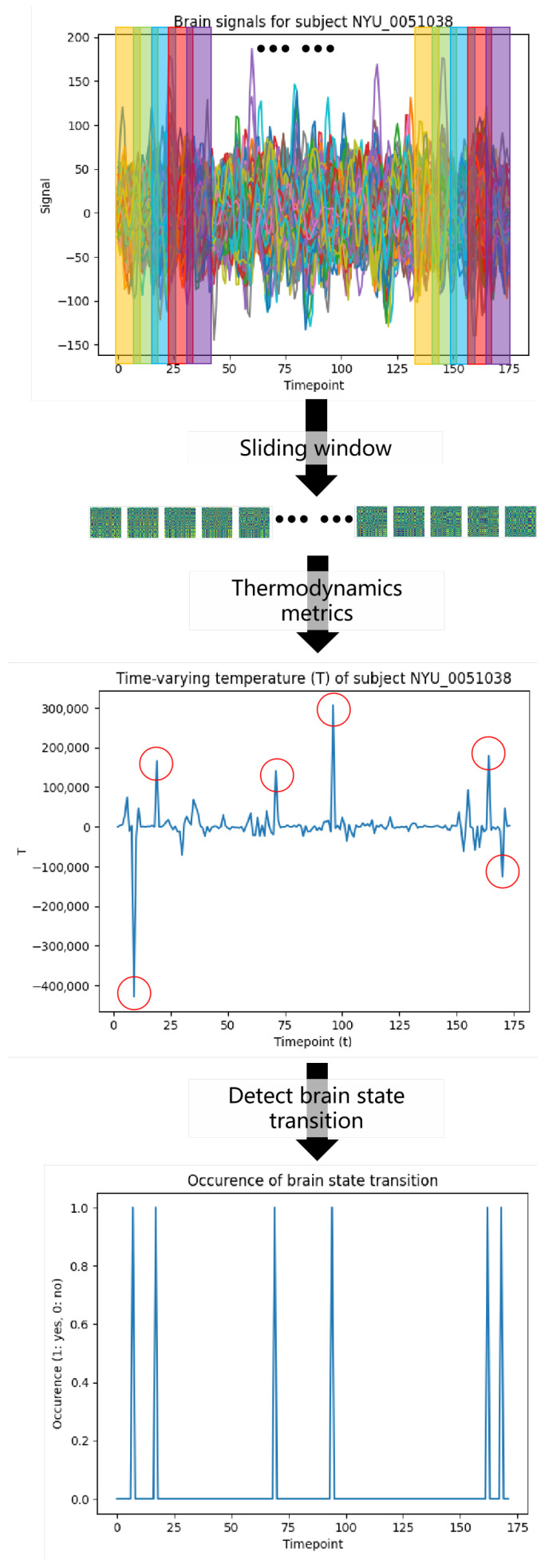
Brain-connectivity matrices are constructed using a sliding-window approach. Thermodynamic metrics are then applied to extract time-varying functional changes and detect brain-state transition points.

With the thermodynamic quantities defined in Section 2, we analyze brain signals through sliding-window network construction, thermodynamic calculation, and statistical correlation testing, as illustrated in Figure 1.

We first adopt a sliding-window approach to extract time-varying functional connectivity from fMRI time series:

- Windowing: Given an fMRI time-series matrix $X\in R^{T\times N}$, where *T* is the number of time points and *N* is the number of brain regions, we segment the data into overlapping windows. Let *w* be the window length and *s* the step size. The *k*-th windowed segment is
  (12) $$X_{k}=X\left[t_{k}:t_{k}+w-1,:\right]$$
  where $t_{k}=k\cdot s$ and $k\in \{0,1,\ldots ,\left\lfloor \frac{T-w}{s}\right\rfloor \}$.
- Connectivity matrix calculation: For each windowed segment $X_{k}$, we compute the connectivity matrix $A_{k}\in R^{N\times N}$ using Pearson correlation, with diagonal elements set to zero:
  (13) $$A_{k}\left(i,j\right)=\left\{\begin{array}{cc} \frac{\mathrm{Cov}(X_{k}\left(:,i\right),X_{k}\left(:,j\right))}{\sigma \left(X_{k}\left(:,i\right)\right)\;\sigma \left(X_{k}\left(:,j\right)\right)}, & i\neq j,\\ 0, & i=j. \end{array}\right.$$

To focus on dominant functional connections, we threshold each connectivity matrix by setting correlations greater than 0.5 to 1 and all others to 0, yielding binary adjacency matrices. We use a fixed threshold to emphasize stronger and more stable functional connections within short sliding windows and to preserve a transparent interpretation of the resulting graph quantities. As thresholding can influence derived connectome properties [32,33,34], the present choice should be viewed as a design decision within the current binary-graph formulation rather than a uniquely optimal value [35].

The resulting adjacency matrices define a time-varying brain network. We then summarize each matrix using the thermodynamic metrics introduced in Section 2. As illustrated in Figure 1, these quantities exhibit spikes at certain time points, suggesting candidate instants of brain-state transition.

To validate whether these thermodynamic changes align with structural reconfiguration in the network, we compare consecutive adjacency matrices using the Structural Similarity Index (SSIM) [36]. Treating adjacency matrices as two-dimensional gray-scale images, SSIM provides an image-based similarity score that jointly reflects luminance, contrast, and structural agreement:

(14) $$\mathrm{SSIM}\left(x,y\right)=\frac{\left(2\mu_{x}\mu_{y}+C_{1}\right)\left(2\sigma_{xy}+C_{2}\right)}{\left(\mu_{x}^{2}+\mu_{y}^{2}+C_{1}\right)\left(\sigma_{x}^{2}+\sigma_{y}^{2}+C_{2}\right)},$$

where $\mu_{x}$ and $\mu_{y}$ are the means of *x* and *y*, $\sigma_{x}^{2}$ and $\sigma_{y}^{2}$ are the corresponding variances, and $\sigma_{xy}$ is their covariance. In our setting, low SSIM values indicate abrupt structural dissimilarity between connectivity matrices from adjacent windows and are therefore used as candidate markers of brain-state transitions.

We calculate the correlation between the occurrences of temperature spikes and low SSIM values using Matthew’s Correlation Coefficient (MCC), which is designed for binary vectors. We then run a permutation test to assess the statistical significance of this correspondence.

### Operational Definition and Lag-Aware Evaluation

At each time index *t*, we compute $T_{t}=\Delta U_{t}/\Delta S_{t}$ and $\mathrm{SSIM}(A_{t},A_{t-1})$. An event label is assigned when SSIM falls below the cutoff $q_{S}\in \left\{0.05,0.07,0.10\right\}$ (empirical quantiles per subject). Here, $T_{t}$ is used as a thermodynamics-inspired reconfiguration index, and we focus on pronounced excursions of this index as markers of stronger changes between adjacent network states. To account for windowing misalignment, performance is evaluated in a lag-aware manner: a positive SSIM event centered at $t^{*}$ is considered correctly detected if a high thermodynamic score occurs within a ±2-window neighborhood of $t^{*}$. We summarize per-subject AUROC and average precision (AP), and report cohort-level medians across subjects and $q_{S}$ cutoffs. The ±2 tolerance is used only for the real-data and surrogate analyses, where event labels are derived from SSIM over overlapping sliding windows. In the synthetic benchmark, where the ground-truth change points are exact segment boundaries, we use a narrower ±1 tolerance.

## 4. Synthetic Validation on Controlled Dynamic Graphs

We first validate the proposed thermodynamics-inspired metrics on controlled synthetic dynamic graphs where the generative mechanisms are fully known. This allows us to test sensitivity to structure-only, strength-only, and mixed reconfiguration under matched conditions, and to compare against a state-of-the-art spectral baseline.

### 4.1. Generative Model and Regimes

Time-varying weighted graphs are generated using a degree-corrected stochastic block model (DC–SBM) [37] with n=80 nodes and k=4 blocks. Each series contains three segments of equal length (15 windows per segment; 45 windows per series), and segment boundaries form the ground-truth change points. We consider three regimes:

- R1: structure-only (rewire). Within/between-community wiring probabilities $\left(p_{\mathrm{in}},p_{\mathrm{out}}\right)$ change across segments while we approximately preserve the total edge mass *U* between successive windows by a light rescaling, hence ΔU≈0.
- R2: strength-only (gain). The connection pattern is held fixed within segments while edge weights are modulated multiplicatively by a global gain, producing ΔU≠0 with small |ΔSCE|.
- R3: mixed. Modest rewiring is combined with gain modulation, yielding both mass and spectral changes.

Unless stated otherwise, we sweep multiple within/between-community probability settings in R1 and multiple gain levels in R2 and R3. The full parameter grids are reported in Appendix B. Five repeats are run per parameter cell. We work with weighted graphs to avoid artifacts due to hard binarization, which would suppress ΔU and trivially degrade *T* in structure-only settings.

### 4.2. Metrics and Baselines

Per window we compute internal energy $U=\sum_{i<j}w_{ij}$ and Spectral Core Entropy (SCE) from the top-α normalized-Laplacian eigenvalues. Numerical inspection showed that no zero or near-zero ΔS values occurred when computing T=ΔU/ΔS, so no clipping, exclusion, or denominator regularization was used. The weighted synthetic formulation is the general version of the graph-energy definition: for weighted graphs, $w_{ij}$ denotes the edge weight and $d_{i}=\sum_{j}w_{ij}$ is the weighted degree, whereas the binary real-data graphs correspond to the special case $w_{ij}\in \left\{0,1\right\}$, for which $U=\sum_{i<j}w_{ij}=\left|E\right|$. In the main synthetic experiments we use α=20, which gave slightly stronger temperature-based discrimination than α=40 while preserving the same qualitative regime-level conclusions. Robustness to α is examined separately. The temperature index is

$$T_{t}\;=\;\frac{\Delta U_{t}}{\Delta S_{t}},$$

with the first time point set to zero. We score reconfiguration by $|T_{t}|$ and $|\Delta {\mathrm{SCE}}_{t}|$.

We compare against two baselines: (i) NetLSD [38], an isomorphism-invariant multi-scale spectral signature based on the heat trace $h\left(t\right)=\sum_{i}e^{-t\lambda_{i}}$; the dynamic score is the $\ell_{2}$ distance $∥h_{t}-h_{t-1}{∥}_{2}$ over a log-spaced grid of *t* values; (ii) SSIM applied to adjacency matrices, reported as $1-\mathrm{SSIM}(A_{t},A_{t-1})$ to indicate dissimilarity.

### 4.3. Event Labeling and Evaluation Protocol

Ground-truth change points are the segment boundaries. To account for windowing misalignment, we allow a ±1 lag tolerance when matching detections to ground truths. We report threshold-free AUROC; results are aggregated as regime-level medians and means over all parameter cells and repeats.

### 4.4. Results

The Monte Carlo results showed in Table 2 support a regime-specific division of labor rather than a single universally dominant indicator. In structure-only reconfiguration (R1), |ΔS| and NetLSD provide the strongest discrimination, while |T| remains near chance as expected because total edge mass is approximately preserved. In strength-only reconfiguration (R2), |T| achieves the strongest overall AUROC, consistent with its role as an energetic-change-per-spectral-core-change index. In the mixed reconfiguration (R3), |ΔS| and NetLSD remain slightly stronger on average, while |T| stays competitive. These results support using |T| when edge mass varies and |ΔS| as a complementary marker of rewiring.

## 5. Experiment on Autism Spectrum Disorder Data

In this section, we examine brain-network activity using the thermodynamics metrics proposed in Section 2. The experiments aim to exemplify the underlying meanings of these thermodynamic metrics in neuroscience scenarios.

### 5.1. Data Acquisition

The experiment data includes the study of time-varying brain connectivity of autism and control subjects. Data were sourced from the NYU section of the ABIDE dataset [39], consisting of 75 participants selected based on data quality. This group includes 40 control subjects and 35 individuals with autism spectrum disorder (ASD).

Each participant underwent a 6 min resting-state fMRI scan with a repetition time (TR) of 2000 ms, yielding 176 time points per subject after processing. The data were preprocessed using the Configurable Pipeline for the Analysis of Connectomes (CPAC) as part of the Preprocessed Connectomes Project, which includes steps such as slice-timing correction, motion correction, skull stripping, and normalization to MNI space.

By using the Nilearn package [40] in Python 3.10, the preprocessed data in the ABIDE dataset [39] can be obtained. The preprocessed dataset version with bandpass filtering, global signal regression, and quality check is available in the data repository, ensuring a higher data quality. In order to extract time series from fMRI data, a further step of registration and masking using a template or atlas is still needed. In this experiment, the template icbm152 is used. These preprocessing steps are crucial in brain data analysis, as factors like individual differences and motion noise can lead to data ambiguity. The NYU and UM_1 subject IDs are provided in Appendix A.

### 5.2. Parcellation and Time-Series Extraction

Regional time series were extracted in MNI space using the HCP–MMP1.0 (Glasser) 360-region parcellation [41]. This choice aligns the node-level analyses (e.g., V2, LO1, STSvp) with well-established cortical areas. Each regional time series was *z*-scored across time prior to connectivity estimation.

### 5.3. Time-Varying Thermodynamics Parameter Curves

Using a sliding-window approach, we can construct the time-varying brain network. For the ABIDE dataset, subjects are typically scanned for a few minutes with around 2 s temporal resolutions. We hence select the window length to be five time instants and the stride to be one time instant, in order to observe finer dynamics in the scale of seconds. In order to capture the predominant interactions and reduce computational complexity, we select the 20 largest eigenvalues (α=20) for SCE computation. Then, we can calculate the thermodynamic parameter value under each window frame.

**Figure 2:**
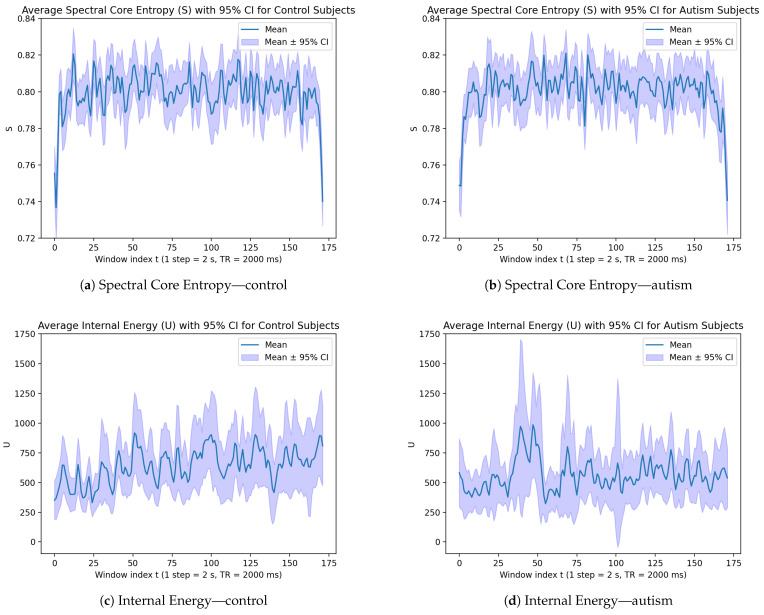

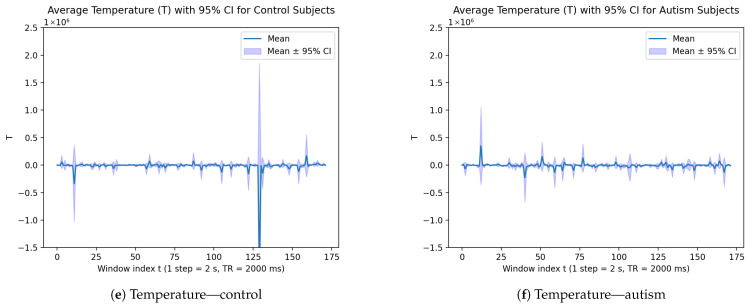
Curves of time-varying entropy, internal energy, and temperature for control and autism groups.

At the group level, we compute the mean value and 95% confidence interval (CI) and plot the curves for entropy, internal energy, and temperature in Figure 2. Both control and autism subjects experience sharp fluctuations in entropy values, indicating that the brains experience a wide variety of different configurations over time. As for the internal energy plots, the autism subjects exhibit more pronounced peaks and wider confidence intervals, suggesting greater variability and possibly more erratic changes in internal energy. The control subjects, while also showing variability, appear to have more stable internal energy with less extreme peaks and narrower confidence intervals. On the temperature plots, the autism subjects show more fluctuations with great spikes in temperature. The control subjects, however, have more consistent temperature measurements with less extreme anomalies. These differences may indicate distinct patterns of internal energy and temperature dynamics between autism and control subjects, which could be relevant for understanding the underlying neurofunctional mechanisms.

**Figure 3:**
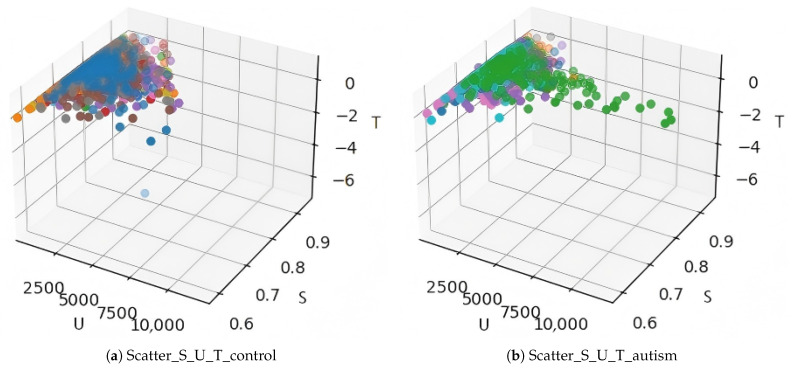
The three-dimensional scatter plot of entropy, internal energy, and temperature at every time point, with each subject marked with a different color.

We can also examine the result by plotting the three-dimensional scatter plot of entropy, internal energy, and temperature at every time point, as shown in Figure 3. It is worth noticing that there are some outliers identifiable in the plot. These outliers are extreme values indicating the time points where transitions of brain states happen.

### 5.4. Lag-Aware, Threshold-Free Evaluation of Reconfiguration Events

We operationalize reconfiguration events as instants where $\mathrm{SSIM}(A_{t},A_{t-1})$ falls below a quantile $q_{S}\in \left\{0.05,0.07,0.10\right\}$. This SSIM-based labeling is independent of our thermodynamic metrics (avoiding circularity). We then treat $|T_{t}|$ and $|\Delta {\mathrm{SCE}}_{t}|$ as continuous scores and evaluate discrimination without selecting thresholds via AUROC (with a ±2-window temporal tolerance). Table 3 reports AUROC medians (with ranges across $q_{S}$). Across window lengths w∈{5,10,15} and both groups, |T| shows robust performance (median AUROC ≈0.79–0.84), while |ΔSCE| provides a complementary, moderate signal (median AUROC ≈0.61–0.67).

### 5.5. Comparison with a Lightweight Clustering Baseline

To position the proposed thermodynamic score relative to a simple brain-state baseline, we applied *k*-means clustering (k=4) to sliding-window correlation matrices and treated cluster-switch instants as candidate transition events. The results are summarized in Table 4. Under the same lag-aware evaluation protocol and the main event setting, this baseline remained close to chance in both groups and substantially below the corresponding thermodynamic event-detection results. This suggests that the proposed index captures reconfiguration information beyond simple clustering switches in correlation space.

### 5.6. Sensitivity Analysis

For clarity of the main narrative, detailed sensitivity analyses for window size and the number of retained eigenvalues α are reported in Appendix C. Overall, the temporal patterns of the thermodynamic quantities remain stable across tested settings. For the event-detection analysis, additional checks over α∈{5,10,20,30} show that the qualitative pattern is preserved across values, although the numerically strongest AUROC differs slightly by group. To avoid group-specific tuning and to maintain a single shared setting throughout the manuscript, we retain α=20 as the main choice.

### 5.7. Top-α Spectral Core and a Renormalization Control

We examine how the top-α spectral core tracks full-spectrum von Neumann entropy (VNE) dynamics. For each subject and window length w∈{5,10,15}, we compute per-window full VNE and SCE with α∈{20,40,60,100} in two ways: (i) the original SCE keeps only the top-α eigenvalues without renormalizing the omitted tail $\left(p_{i}=\lambda_{i}/V\right)$, summarizing core spectral concentration; (ii) a renormalized control rescales within the top set, $p_{i}^{\left(\alpha \right)}=\lambda_{i}/\sum_{\mathrm{top}-\alpha }\lambda$, measuring within-core dispersion.

To focus on temporal reconfiguration rather than absolute levels, we use first differences. Let ${\left\{{\mathrm{VNE}}_{t}\right\}}_{t=1}^{T_{w}}$ and ${\left\{{\mathrm{SCE}}_{t}\right\}}_{t=1}^{T_{w}}$ be the per-window series; we define

$$\Delta {\mathrm{VNE}}_{t}:={\mathrm{VNE}}_{t}-{\mathrm{VNE}}_{t-1},\;\Delta {\mathrm{SCE}}_{t}:={\mathrm{SCE}}_{t}-{\mathrm{SCE}}_{t-1},\;t=2,\ldots ,T_{w}.$$

We then compute Spearman’s rank correlation as

$$\rho \;=\;{\mathrm{corr}}_{\mathrm{Spearman}}\;\left({\left\{\Delta {\mathrm{VNE}}_{t}\right\}}_{t=2}^{T_{w}},\;{\left\{\Delta {\mathrm{SCE}}_{t}\right\}}_{t=2}^{T_{w}}\right),$$

and summarize cohort medians.

Consistent with design, the original SCE is anti-aligned with full-spectrum dispersion (median ρ<0 for all w,α), because shifting spectral mass toward leading modes reduces global dispersion while increasing core concentration. The renormalized control flips the sign and shows stronger positive alignment as α grows at short windows (e.g., w=5,α=100: Control $\rho_{\mathrm{median}}=0.699$; ASD $\rho_{\mathrm{median}}=0.694$), indicating that dominant modes carry most of the dynamic signal. Complete grids appear in Appendix D.

### 5.8. Analysis of Subject-Level Metrics Across Groups

To avoid distortions induced by direct group-wise time-series averaging, we summarize each subject by the standard deviation of internal energy and entropy and then compare autism and control groups at the subject level. We use the Kolmogorov–Smirnov (KS) test for group comparison and report Kullback–Leibler (KL) divergence only as a descriptive complement.

The results are presented in Table 5. These results demonstrate significant group differences in the standard deviation of internal energy. For the standard deviation of entropy, the KS test does not reach the conventional significance threshold, although the KL divergence remains relatively large, suggesting a potentially meaningful but weaker distributional difference.

These findings suggest that the variability captured by subject-level thermodynamic summaries is informative for distinguishing neurofunctional dynamics between groups.

Building on this observation, we next focus on the temperature metric, which is derived from the relationship between changes in internal energy and entropy over time. We examine whether temperature spikes align with candidate brain-state transitions and assess their neurofunctional significance.

### 5.9. Temperature Spikes and Brain State Transitions

By its definition and mathematical formulation, temperature symbolizes the structural change between the systems at *t* and t−1 time instants. In brain-network analysis, this implies critical events such as brain-state transitions [42].

**Figure 4:**
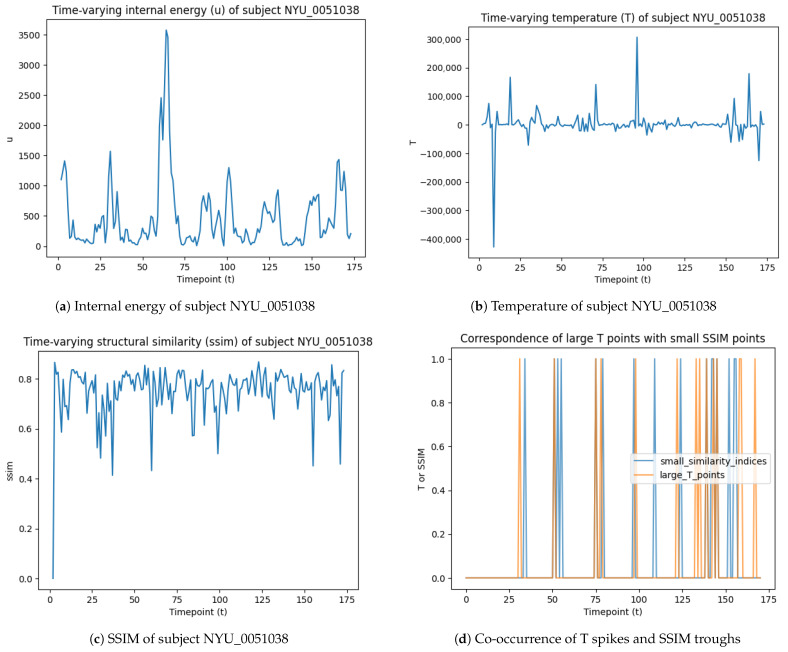
Illustration of time-varying physical quantities and brain-state transition detection of subject NYU_0051038.

Using the subject NYU_0051038 as an example, we show individual-level analysis of temperature spikes and their relation to the brain-state transition. The internal energy and temperature of NYU_0051038 are illustrated in Figure 4. The troughs in the SSIM plot suggest low similarities between the brain patterns at the past two time points, hence identifying changes in the brain state. We show here that the T spikes and SSIM troughs of the subject NYU_0051038 correspond very well, motivating the following analysis of the statistical relationship between these two phenomena.

### 5.10. Statistical Testing for T Spikes and SSIM

To generalize the finding of the correspondence between T spikes and SSIM troughs of the subject NYU_0051038 to the group level, we test the MCC correlation between the occurrence of large temperature points and low structural similarity values and run a permutation test to determine the likelihood of obtaining a correlation value that is equally or more extreme. We summarize the average MCC and p-value of autism and control groups in Table 6. Because permutation tests were performed separately for each subject, the group-level *p*-value summaries are interpreted descriptively rather than as formal group-level hypothesis tests. We therefore report both the mean and median subject-level *p*-values, together with the interquartile range, to summarize the consistency of the evidence across subjects.

Although the average MCC values are modest (around 0.24–0.25), the associated permutation-test *p*-values indicate that this correspondence is unlikely to arise from chance alone. We therefore interpret temperature spikes not as a highly accurate event detector by themselves, but as a weak yet reproducible marker of rapid network reconfiguration. This interpretation is further supported by the following preliminary UM_1 cross-site validation, which retains positive MCC values in both groups, and by the edge-count-preserving surrogate null, under which the correspondence collapses to near-chance levels.

### 5.11. Preliminary Cross-Site Validation

To assess whether the event-level findings generalize beyond the NYU site, we further conducted a preliminary cross-site validation on an independent UM_1 subset of ABIDE dataset containing 15 ASD subjects and 15 control subjects. The results are summarized in Table 7. Using the same experimental configuration as in the main analysis, we computed the average Matthews Correlation Coefficient (MCC) and the corresponding permutation-test *p*-values for the association between temperature spikes and low-similarity events.

Although this cross-site cohort is relatively small, the positive MCC values and statistically significant permutation-test results in both groups provide preliminary evidence that the association identified at NYU is not entirely site-specific.

### 5.12. Edge-Count-Preserving Surrogate Null

To test whether the observed correspondence can be explained by edge count alone, we constructed an edge-count-preserving surrogate null under the same main analysis setting (window size =5, threshold =0.5, α=20, and lag tolerance ±2). For each sliding window, we preserved only the number of nonzero edges in the binarized graph and randomly reassigned their positions, thereby destroying higher-order structural organization while keeping edge count unchanged. We then recomputed the temperature series on the surrogate graph sequence and evaluated it against the same low-SSIM event labels used in the main NYU analysis. The results are summarized in Table 8.

Under this surrogate null, performance collapses to near-chance levels in both groups, indicating that the observed event correspondence is not reproduced by edge count alone. This suggests that the proposed temperature index is sensitive to the structural arrangement of connections rather than merely to fluctuations in graph density.

## 6. Node Energy Observations and Group Differences

**Figure 5:**
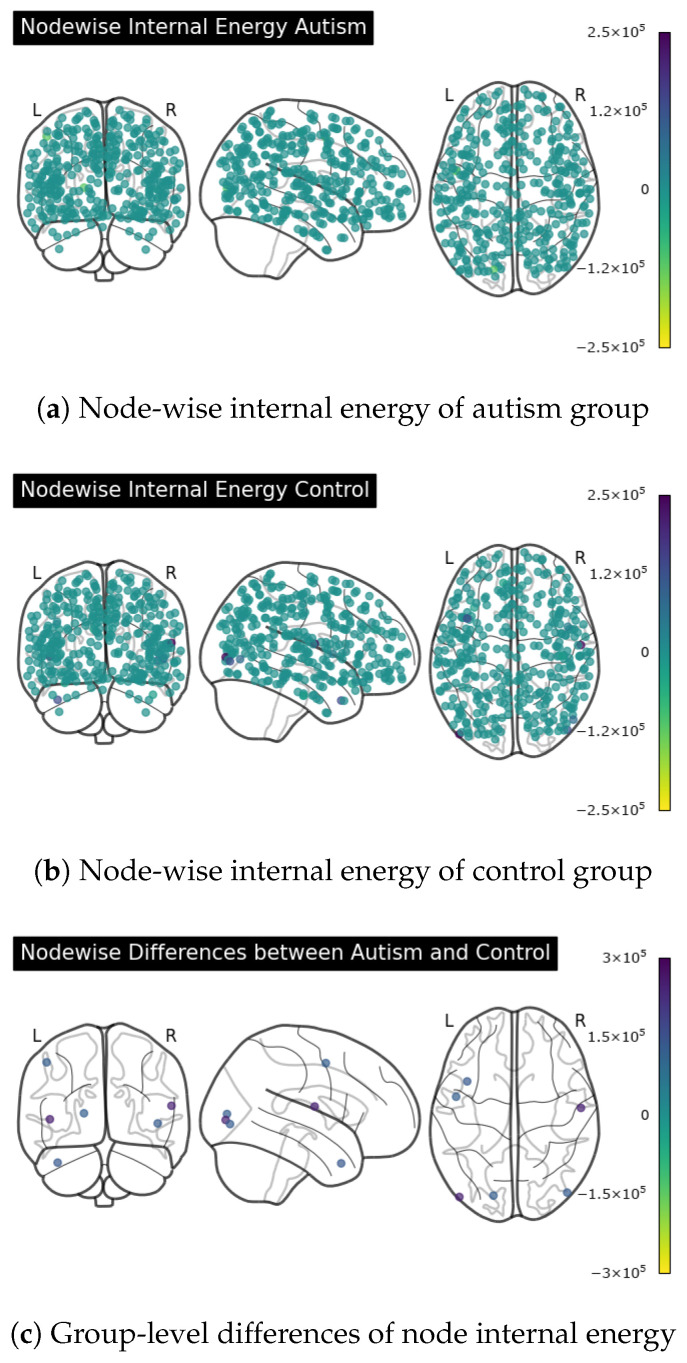
Node-level internal energy and group-level differences.

We compute the groups node energy for each node, as visualized in Figure 5. In our experiments, since each node represents a corresponding brain region, node energy is an important quantity reflecting the activity level of that brain region within the network. We evaluated node energy across all 360 Glasser regions and calculated the differences between autism and control subjects, finding clear discrepancies for several regions. We illustrate the nodes with the most prominent differences in Figure 5c, where notable differences are found for nodes 4, 11, 20, and 200. According to the Glasser atlas labels [41], the regions listed in Table 9 correspond to those showing the largest absolute ASD–control node-energy differences and are presented here as descriptive observations for an explanatory neuroanatomical context. We identified the corresponding brain regions and found relevant biological studies reporting autism-related differences in similar functional systems. As a preliminary cross-site check, 7 of the top 10 NYU regions ranked by absolute ASD–control node-energy differences showed the same ASD–control direction in the independent UM_1 subset, suggesting that the strongest effect-ranked regions are not entirely site-specific.

According to Table 9, indices 4, 11, 20, and 200 correspond to regions associated with visual functions, suggesting altered visual-related signal organization in the ASD group. These observations are consistent with prior studies reporting atypical visual and social-perceptual processing in autism [43], which found that individuals with autism may show atypical processing of other people’s emotions and facial expressions, resulting in deteriorated levels of empathy and social ability. Region 130 is also about social perceptions and language, and it is a part of the default mode network (DMN), which is active during rest and involved in self-referential thinking, mind-wandering, and autobiographical memory. The default mode network (DMN) denotes a large-scale network typically engaged during rest and internally directed cognition and here it is used as an anatomical–functional label to situate regions (e.g., superior temporal sulcus) within known networks [44]. Differences related to region 130 could be linked to internally directed cognition and higher information density produced by autistic brains at rest [45]. Studies have also shown altered language development processes among subjects with autism [46]. There are also studies showing altered DMN functions and their relation with social withdrawal in autism [44], as well as atypical linkage mechanisms among visual, motor, and DMN regions [47]. Taken together, these prior findings provide plausible neuroscientific context for interpreting the observed node-energy differences, although the present study does not by itself establish the underlying biological mechanism. Overall, these results suggest that node energy may serve as an interpretable node-level descriptor for summarizing regional differences in dynamic brain connectivity, showing the potential to serve as a node feature to represent the network.

### Scope Note

DMN-related statements in this work are framed as qualitative contextualization. Formal tests of DMN-specific hypotheses will be pursued with longer acquisitions, harmonized preprocessing, and targeted DMN parcellations.

## 7. Conclusions and Future Work

In this paper, we proposed a thermodynamics-inspired framework for characterizing reconfiguration in dynamic complex networks, with application to resting-state brain connectivity in autism spectrum disorder. The framework is centered on Spectral Core Entropy (SCE), node energy, graph internal energy, and a temperature-like reconfiguration index, which together provide a compact representation of graph interaction mass, spectral organization, and their coupled changes over time.

From a methodological perspective, the main contribution of this work is not only the introduction of these quantities individually, but also their integration into a unified representation for dynamic graph analysis. In particular, SCE provides a tractable spectral descriptor of meso-scale structural reconfiguration, node energy offers an interpretable node-level summary of local graph participation, and the temperature index links energetic and spectral variation across adjacent graph states.

The experimental results support the usefulness of this framework at multiple levels. On controlled synthetic dynamic graphs, the proposed quantities exhibit complementary regime sensitivity, showing that spectral-core change is more informative for rewiring-dominated changes, whereas the temperature index is more informative for gain-dominated changes. On ABIDE–NYU rs-fMRI, the temperature index shows a statistically significant but modest association with low-SSIM reconfiguration events, indicating that it acts as a weak yet reproducible marker of rapid network reorganization, with the overall findings remaining robust and interpretable under additional evaluation. At the node level, node-energy differences highlight visual and superior temporal/default-mode-related regions, providing plausible neuroscientific context while not by themselves establishing biological mechanism.

Overall, the proposed framework offers a novel and computationally tractable way to represent dynamic graph reconfiguration, and its utility is not limited to brain connectivity alone. Future work will extend this formulation to weighted dynamic graphs, broader multi-site and multi-modal datasets, and other evolving complex-network settings such as financial and relational systems.

## Figures and Tables

**Table 1:** Mapping between classical thermodynamic terms and graph-information counterparts used in this work.

Classical Term	Graph/Information Counterpart in This Paper
Micro-states	Normalized-Laplacian eigenmodes
State probabilities	$p_{i}={\tilde{\lambda }}_{i}/\sum_{j}{\tilde{\lambda }}_{j}={\tilde{\lambda }}_{i}/\left|V\right|$
Spectral Core Entropy *S*	*S* keeps top-α modes of von Neumann entropy
Internal energy *U*	Edge mass; discretely $U=\sum_{i}U_{i}=\left|E\right|$ under our locality-based node energy
Temperature *T*	Discrete reconfiguration index T=ΔU/ΔS between adjacent windows
Isolated system	A short sliding window treated as a closed accounting unit (node set fixed)

**Table 2:** Synthetic validation: per-regime AUROC (median/mean) aggregated over 500 Monte Carlo grid sweeps.

Regime	|T|	|ΔS|	NetLSD	1−SSIM
R1 (structure-only)	0.474/0.482	**0.585/0.586**	**0.585/0.586**	0.568/0.571
R2 (strength-only)	**0.560/0.554**	0.513/0.515	0.513/0.516	0.517/0.516
R3 (mixed)	0.556/0.553	**0.590/0.587**	0.583/0.586	0.577/0.578

**Table 3:** Lag-aware, threshold-free detection of low-SSIM reconfiguration events. Entries show median AUROC [min–max] across $q_{S}\in \left\{0.05,0.07,0.10\right\}$.

Group	Window (*w*)	|T| AUROC (Median [Range])	|ΔSCE| AUROC (Median [Range])
Control	5	0.806 [0.798–0.816]	0.609 [0.577–0.629]
ASD	5	0.814 [0.814–0.826]	0.566 [0.545–0.587]
Control	10	0.786 [0.759–0.813]	0.635 [0.632–0.640]
ASD	10	0.825 [0.799–0.837]	0.638 [0.617–0.642]
Control	15	0.806 [0.771–0.807]	0.671 [0.645–0.688]
ASD	15	0.792 [0.789–0.809]	0.637 [0.620–0.648]

**Table 4:** Lightweight *k*-means baseline (k=4) under the main evaluation setting.

Group	*MCC*_median_	AUROC_median_	AP_median_
ASD	0.0284	0.5071	0.2840
Control	0.0099	0.5026	0.2931

**Table 5:** Results of Kolmogorov–Smirnov (KS) tests and Kullback–Leibler (KL) divergence for group-wise comparisons.

Metric	KS Statistic (*p*-Value)	KL Divergence
Standard Deviation of Internal Energy	0.327 (p=0.042)	7.841
Standard Deviation of Spectral Core Entropy	0.302 (p=0.071)	12.206

**Table 6:** MCC and subject-level permutation-test summaries for autism and control groups.

Group	${\mathrm{MCC}}_{\mathrm{avg}}$	*p*-Value_avg_	$p_{\mathrm{median}}$	$p_{\mathrm{IQR}}$
Autism	0.238	0.0254	0.0150	0.0035–0.0345
Control	0.252	0.0224	0.0065	0.0018–0.0297

**Table 7:** Preliminary cross-site validation on the UM_1 subset (15 ASD and 15 control subjects).

Group	${\mathrm{MCC}}_{\mathrm{avg}}$	*p*-Value_avg_
Autism	0.1518	0.0147
Control	0.1625	0.0322

**Table 8:** Edge-count-preserving surrogate null under the main NYU analysis setting.

Group	${\mathrm{MCC}}_{\mathrm{median}}$	$p_{\mathrm{median}}$	AUROC_median_
Autism	−0.0046	0.6424	0.4788
Control	−0.0100	0.6603	0.5109

**Table 9:** Brain regions with most prominent differences and their corresponding functions.

Index	Label	Brain Region	Function
4	Right_V2	secondary visual cortex	visual information processing
11	Right_PEF	parietal eye field	dorsal attention and eye movement
20	Right_LO1	lateral occipital area 1	visual perception (object recognition, motion detection)
130	Right_STSvp	superior temporal sulcus	default mode network, rest activities, auditory and social perception
200	Left_LO1	lateral occipital area 1	visual perception (object recognition, motion detection)
279	Left_43	primary gustatory cortex	somatosensory, taste, and mouth functions

**Figure A1:**
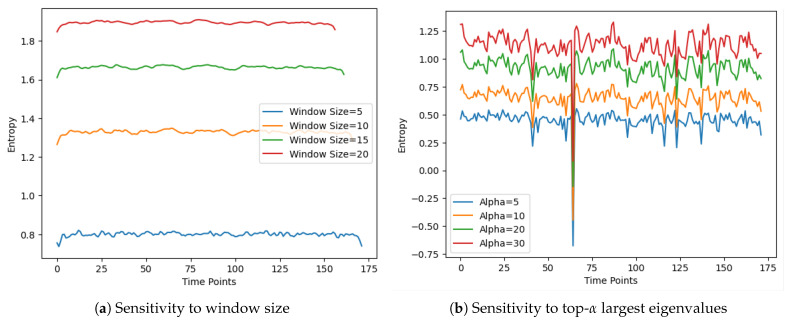
Sensitivity analysis of entropy with respect to (**a**) window size and (**b**) the number of retained eigenvalues (α). Absolute levels change as expected, while temporal patterns remain stable.

## Data Availability

Data available in a public repository. The data presented in this study are openly available from the Autism Brain Imaging Data Exchange (ABIDE) at https://fcon_1000.projects.nitrc.org/indi/abide/, accessed on 15 March 2024.

## Appendix A. Subject-Selection Criteria and Subject IDs

For reproducibility, we report the subject-selection criteria used in the NYU main analysis and UM_1 preliminary cross-site validation. For both sites, subjects were included if CPAC-preprocessed, quality-checked resting-state fMRI data were available, the time series were complete for the analysis, HCP–MMP1.0 regional time-series extraction was successful, and the resulting regional signals and connectivity matrices were valid for the subsequent thermodynamic calculations. Subjects for which regional time-series extraction or connectivity-matrix construction failed were not included. Diagnostic labels were taken from the ABIDE phenotypic metadata.

The NYU analysis included 75 subjects, consisting of 40 control subjects and 35 ASD subjects. The selected NYU participants follow the ABIDE site-specific subject-ID format NYU_005XXXX.

The UM_1 preliminary cross-site validation used an independent balanced subset of 30 subjects, consisting of 15 control subjects and 15 ASD subjects. No model parameter, graph threshold, event threshold, or lag tolerance was tuned on the UM_1 subset. The selected UM_1 participants follow the ABIDE site-specific subject-ID format UM_1_005XXXX. The exact subject IDs used in the analyses are available from the corresponding author upon reasonable request.

## Appendix B. Synthetic Parameter Grids

The synthetic experiments used a weighted degree-corrected stochastic block model (DC–SBM) with n=80 nodes and k=4 blocks. Each dynamic graph series contained three segments, with 15 windows per segment and 45 windows in total. Ground-truth change points were defined at the segment boundaries, and a ±1-window lag tolerance was used for event matching.

The parameter grid contained 16 cells in total: 9 cells for R1, 3 cells for R2, and 4 cells for R3. For each parameter cell, we generated five independent repeats. Therefore, for a fixed spectral-core size and temperature mode, each Monte Carlo grid sweep contained 16×5=80 synthetic dynamic-graph series. The reported synthetic results were aggregated over 500 Monte Carlo grid sweeps with different base seeds. The tested spectral-core sizes were α∈{20,40}.

**Table A1:** Parameter grids used in the synthetic dynamic-graph experiments.

Regime	Parameter Grid	Number of Cells
R1 structure-only	$p_{\mathrm{in}}\in \left\{0.12,0.14,0.16\right\}$, $p_{\mathrm{out}}\in \left\{0.03,0.04,0.05\right\}$	3×3=9
R2 strength-only	gain∈{0.7,1.3,1.6}	3
R3 mixed	$\left(p_{\mathrm{in}},p_{\mathrm{out}}\right)\in \left\{\left(0.12,0.03\right),\left(0.16,0.05\right)\right\}$, gain∈{1.2,1.5}	2×2=4

## Appendix C. Sensitivity Analysis

### Appendix C.1. Effect of Window Size

We examined window sizes w∈{5,10,15,20} with a stride of 1. Increasing the window length raises the absolute entropy values because larger windows aggregate more temporal information and tend to produce smoother adjacency matrices and more uniform eigenvalue spectra. Importantly, the temporal patterns of entropy remain stable across window sizes, indicating that the main conclusions about reconfiguration dynamics are not tied to a single window choice. Because the full time series contains only T=176 time points, very large windows also reduce the number of valid windows and may therefore be less desirable for short acquisitions.

### Appendix C.2. Effect of the Retained Spectral-Core Size α

We varied the number of retained eigenvalues α∈{5,10,20,30} when computing SCE. As expected, retaining more eigenvalues changes the absolute entropy level because a larger portion of spectral mass contributes to the calculation, whereas smaller α values yield lower absolute values. Nevertheless, the relative temporal trajectories remain qualitatively stable, showing that the framework captures consistent reconfiguration patterns over a broad range of α.

For the event-detection analysis, we additionally performed an auxiliary α-sensitivity check under a single event setting, with lag tolerance implemented by event-label dilation. This analysis is intended to examine the qualitative effect of varying α, rather than to reproduce the across-$q_{S}$ best-lag AUROC summary reported in Table 3. The qualitative discrimination pattern is preserved across tested values, but the numerically strongest AUROC differs slightly by group: the control group peaks at α=10, whereas the ASD group peaks at α=20. To avoid group-specific tuning and preserve a single shared configuration throughout the manuscript, we retain α=20 as the main setting.

**Table A2:** Auxiliary α-sensitivity check for AUROC with event-label dilation.

α	Control AUROC	ASD AUROC
5	0.721	0.750
10	**0.767**	0.770
20	0.724	**0.815**
30	0.677	0.775

## Appendix D. Additional Evaluation: Dynamic Alignment Between Δ VNE and ΔSCE

Table A3 and Table A4 report cohort-level medians and means of Spearman’s ρ between ΔVNE and ΔSCE across window lengths and α for the original (non-renormalized) SCE and the renormalized control.

**Table A3:** Control cohort: Spearman ρ between ΔVNE and ΔSCE across window *w* and α.

*w*	α	$\rho_{\mathrm{med}}$ (Original)	$\rho_{\mathrm{med}}$ (Renorm)	$\rho_{\mathrm{mean}}$ (Original)	$\rho_{\mathrm{mean}}$ (Renorm)
5	20	−0.808	0.086	−0.800	0.089
5	40	−0.804	0.363	−0.800	0.362
5	60	−0.813	0.531	−0.810	0.530
5	100	−0.854	0.699	−0.851	0.698
10	20	−0.738	−0.516	−0.738	−0.532
10	40	−0.897	−0.584	−0.893	−0.585
10	60	−0.922	−0.525	−0.917	−0.541
10	100	−0.938	−0.293	−0.936	−0.292
15	20	−0.704	−0.070	−0.705	−0.067
15	40	−0.847	−0.108	−0.843	−0.124
15	60	−0.901	−0.118	−0.898	−0.116
15	100	−0.939	−0.024	−0.938	−0.018

**Table A4:** ASD cohort: Spearman ρ between ΔVNE and ΔSCE across window *w* and α.

*w*	α	$\rho_{\mathrm{med}}$ (Original)	$\rho_{\mathrm{med}}$ (Renorm)	$\rho_{\mathrm{mean}}$ (Original)	$\rho_{\mathrm{mean}}$ (Renorm)
5	20	−0.809	0.081	−0.799	0.080
5	40	−0.809	0.350	−0.800	0.347
5	60	−0.829	0.518	−0.812	0.522
5	100	−0.866	0.694	−0.852	0.692
10	20	−0.727	−0.551	−0.729	−0.538
10	40	−0.894	−0.583	−0.893	−0.590
10	60	−0.914	−0.542	−0.915	−0.550
10	100	−0.933	−0.312	−0.935	−0.310
15	20	−0.712	−0.046	−0.702	−0.057
15	40	−0.851	−0.112	−0.840	−0.111
15	60	−0.903	−0.126	−0.896	−0.116
15	100	−0.938	0.008	−0.936	−0.011
